# Totally Laparoscopic Whipple’s Operation: Initial Report from the Caribbean

**DOI:** 10.7759/cureus.7401

**Published:** 2020-03-24

**Authors:** Yardesh Singh, Shamir O Cawich, Sidiyq Mohammed, Thivy Kuruvilla, Vijay Naraynsingh

**Affiliations:** 1 Surgery, University of the West Indies, St. Augustine, TTO; 2 Surgery, Medical Associates Hospital, St. Joseph, TTO; 3 Clinical Surgical Sciences, University of the West Indies, St. Augustine, TTO

**Keywords:** whipple, pancreas, laparoscopic, mis, pancreaticoduodenectomy

## Abstract

Oncologic surgery in the Caribbean has evolved over the past decade, with increasing reports of advanced minimally invasive operations being performed. However, the minimally invasive approach has not been used for peri-ampullary lesions. This is because a laparoscopic Whipple’s operation is a technically demanding and time-consuming operation. We report the first case of a totally laparoscopic Whipple’s operation to be performed in the Caribbean.

## Introduction

Oncologic surgery in the Caribbean has evolved over the past decade, with increasing reports of advanced minimally invasive operations being reported in the region [1-3]. However, as it relates to pancreatic surgery, the minimally invasive approach has been limited to operations for pancreatic pseudocysts and distal pancreatectomies, with little application to peri-ampullary lesions [4-6]. This is because a totally laparoscopic Whipple’s operation is a technically demanding and time-consuming operation.

We report the case of a totally laparoscopic Whipple’s operation completed in Trinidad & Tobago. To the best of our knowledge, this is the first reported case of a laparoscopic Whipple’s operation to be performed in the Caribbean.

## Case presentation

**Figure 1:**
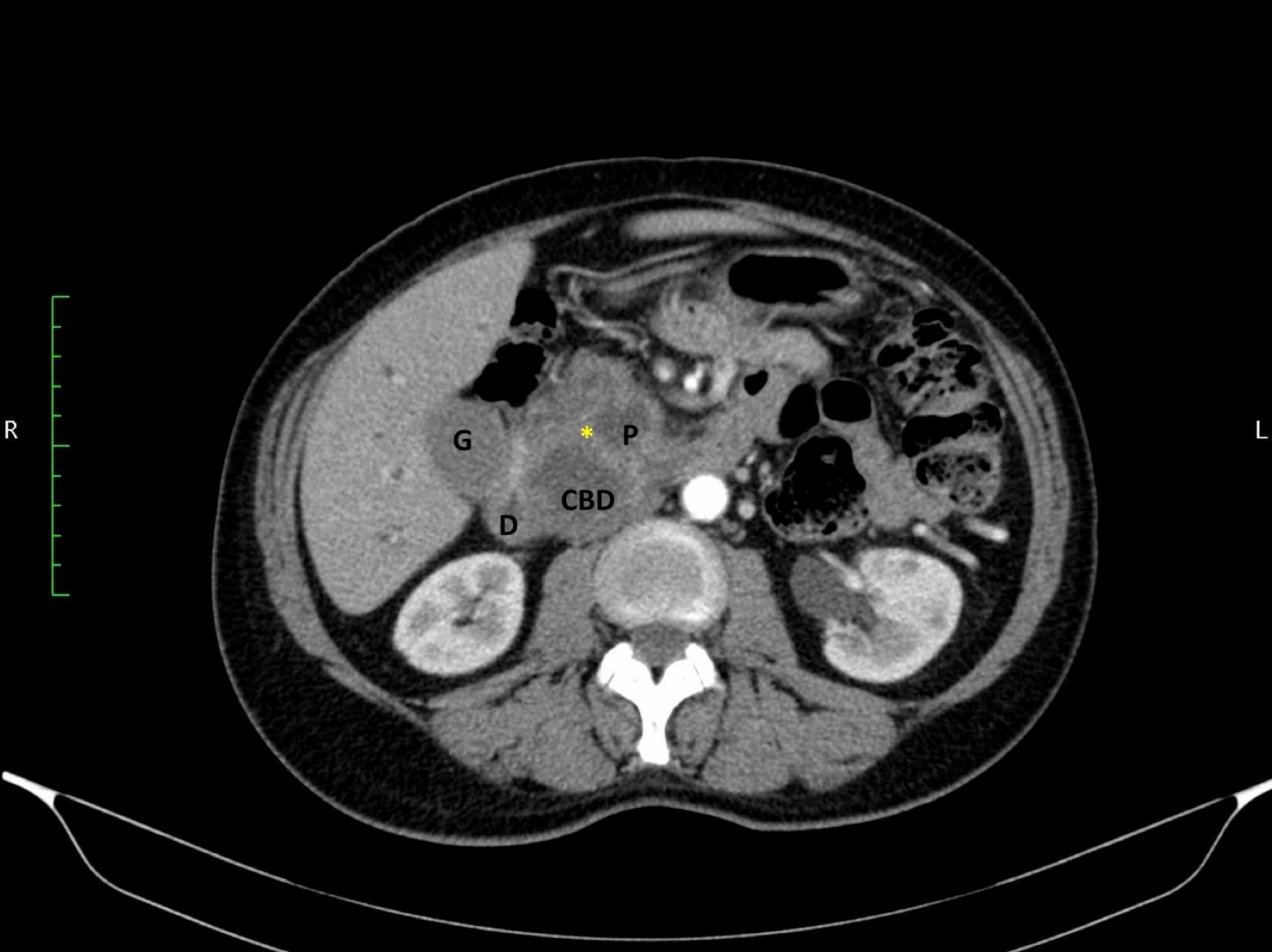
Axial CT scan images demonstrate a heterogeneous mass in the pancreatic head (asterisk) The common bile duct (CBD) and pancreatic duct (P) are also visible, forming a double duct sign. The dilated gallbladder (G) and duodenum (D) are also seen.

A 65-year-old man with no comorbidities presented with a four-week history of progressive jaundice and un-quantified weight loss. Biochemical investigations confirmed direct hyperbilirubinemia and contrast-enhanced computerized tomography scan revealed a heterogenous pancreatic head mass (Figure 1). The common bile duct measured 1.1 cm in diameter and the pancreatic duct measured 1.2 cm in diameter. A preoperative diagnosis of a malignant pancreatic head neoplasm was made. This patient was prepared for general anesthesia. A Whipple’s operation was planned and informed consent was secured to attempt this operation using the laparoscopic approach.

**Figure 2:**
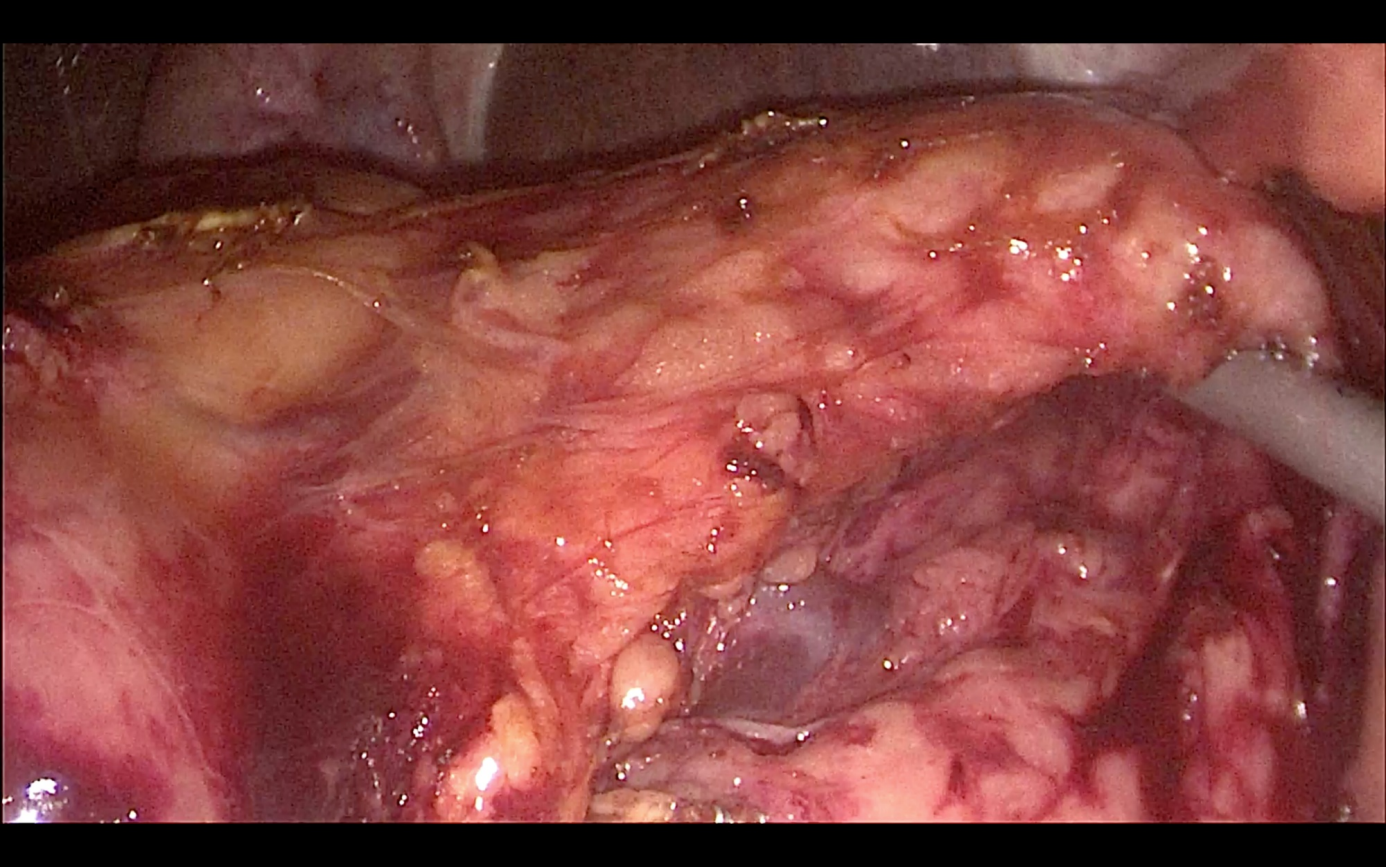
The pancreatic neck is lifted to create a retro-pancreatic tunnel

The patient was placed in the lithotomy position and a five-port technique was used to access the peritoneal cavity. The operation commenced with the division of the gastro-colic ligament to enter the lesser sac, using the Ligasure 5 mm dolphin tip vessel sealer (Medtronic, Minneapolis). The pancreatic neck was identified and a retro-pancreatic tunnel was created under laparoscopic vision (Figure 2). This was achieved using blunt dissection by opening the jaws of the Ligasure dolphin tip vessel sealer under direct vision with a 10 mm 30-degree laparoscope.

The vasculature was well visualized and easily preserved during the creation of the retro-pancreatic tunnel. The pancreatic neck was not transected at this stage. This step was reserved once a decision on resectability was definitively made. Laparoscopic vision allowed the surgeon to perform a Kocher’s maneuver, using laparoscopic forceps to lift the duodenum and a Ligasure 5 mm dolphin tip vessel sealer to dissect the peritoneal reflection. The Ligasure 5 mm dolphin tip vessel sealer was also used to facilitate the dissection of the hepatoduodenal ligament, preserving the proper hepatic artery and portal vein. Once the lesion was deemed resectable, the common hepatic duct was transected with scissors, a Harmonic scalpel (Ethicon Endo-Surgery, Ohio) was used to transect the pancreatic neck and Endo-GIA (Medtronic, Minneapolis) 60 mm medium-thick staples were used to transect the stomach and jejunum.

**Figure 3:**
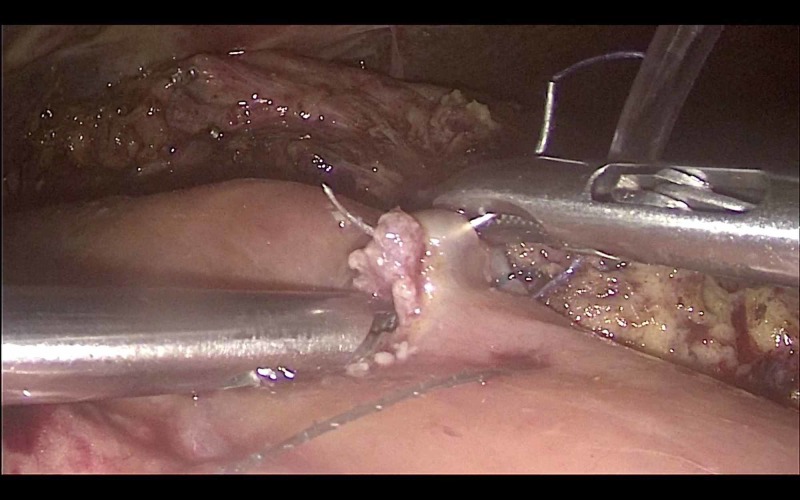
Creation of a sutured hepaticojejunostomy

**Figure 4:**
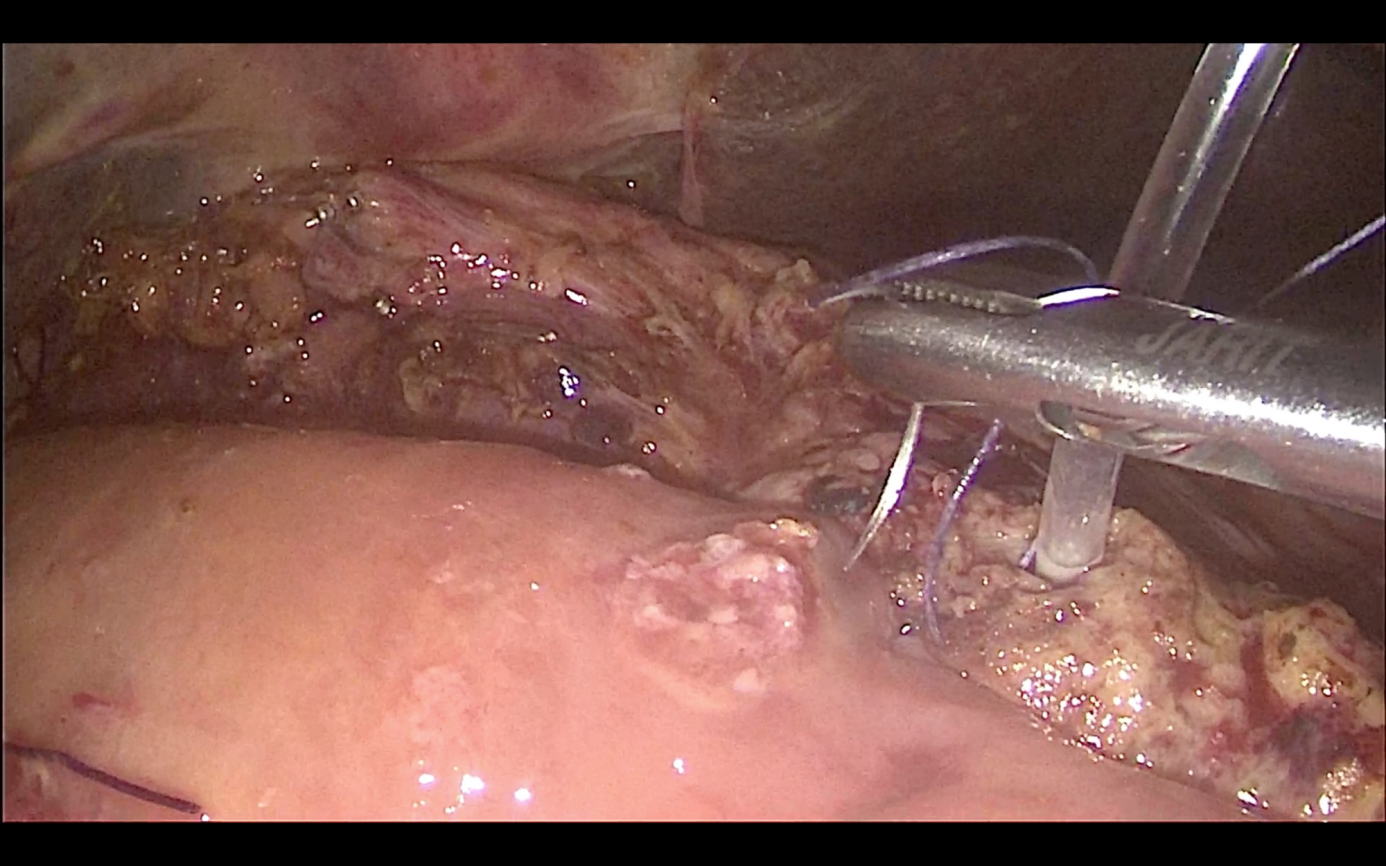
Creation of a sutured pancreaticojejunostomy

**Figure 5:**
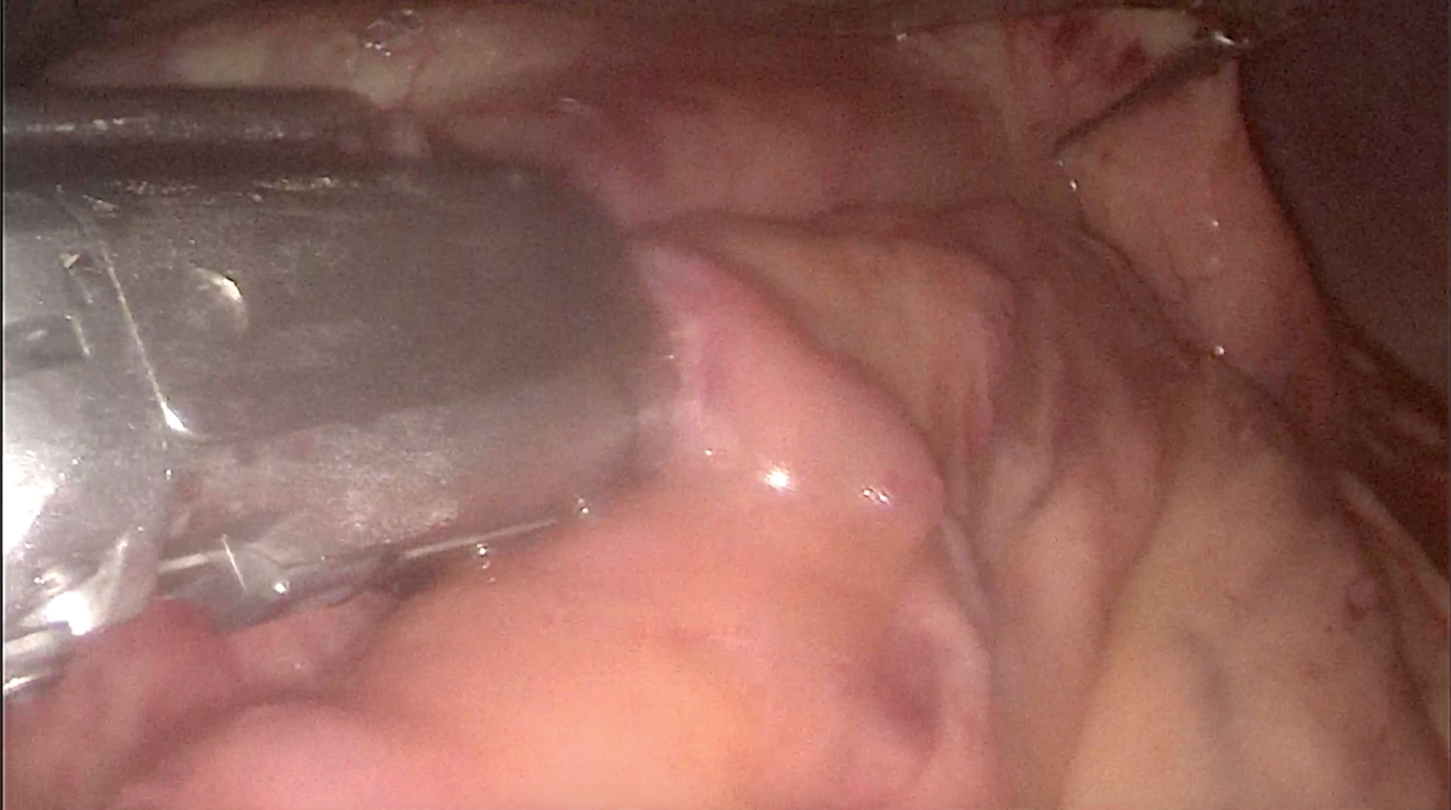
Creation of a stapled gastrojejunostomy

Reconstruction was achieved by a sutured hepaticojejunostomy (Figure 3) using 3/0 PDS sutures in an interrupted fashion. A sutured pancreaticojejunostomy (Figure 4) was created using 3/0 polydioxanone (PDS) sutures in an interrupted technique. Prior to the closure of the pancreaticojejunostomy, a 5Fr infant feeding tube was inserted into the pancreatic duct and a single 3/0 Vicryl suture was placed through the pancreatic duct mucosa and used to fixate the stent, which was left in place. The gastrojejunostomy (Figure 5) and jejunojejunostomy were competed using 60 mm Endo-GIA staples to create a side-to-side anastomosis. The specimen was removed through an extension of the umbilical incision.

The totally laparoscopic Whipple’s operation was completed in a total operating time of 502 minutes, with 200 ml recorded blood loss and no reported complications. This patient was discharged from the high dependency unit within 24 hours of operation. He was ambulating without assistance on Day 3 and discharged from hospital on Day 5 post-operation.

Histologic examination of the specimen confirmed complete resection of an intraductal papillary mucinous neoplasm with low-grade dysplasia. Ten lymph nodes were harvested and all were free of metastatic disease.

## Discussion

A totally laparoscopic Whipple’s operation is arguably one of the most technically complex surgical procedures in existence. Since Gagner and Pomp performed the first totally laparoscopic Whipple’s operation in 1994, it has been performed with increasing frequency across the globe [7]. Six recent metanalyses that compared open versus laparoscopic Whipple’s operations have shown that the operations are oncologically equivalent [8-13]. However, the laparoscopic approach to a Whipple’s operation brings the advantage of reduced physiologic disturbance, rapid recovery, and reduced hospitalization [8-13].

To the best of our knowledge, this is the first report of a totally laparoscopic Whipple’s operation to be performed in the Caribbean. The operation was completed with morbidity and mortality profiles matching those from high-volume centers performing these operations in developed countries [14-16]. It was also accompanied by oncologic outcomes comparable to those from open operations in Caribbean centers of excellence and from high-volume centers in developed countries [17-20].

It was also notable the operation was completed solely by advanced laparoscopic surgeons trained in Caribbean centers. This is testimony to the maturation of minimally invasive surgery training in Caribbean institutions. It also serves as an intrinsic motivator for other surgeons and sets a new standard for surgical oncology in the Caribbean setting.

It is well-accepted that many Caribbean health care systems are economically challenged and resource-poor. This has been one of the limitations in the advancement of minimally invasive surgical practice. However, we have shown that laparoscopic Whipple’s operations are still feasible in this resource-poor environment.

## Conclusions

Despite operating in an under-funded environment, we can incorporate laparoscopic Whipple’s operations into our surgical armamentarium using existing hardware. These procedures should be performed by surgeons with advanced laparoscopic skill sets and considerable experience in pancreatic surgery.
